# Reassessing Global Field Synchronization as a Phase‐Synchrony EEG Metric: Mathematical Review and Empirical Counterexamples With Implications for Wearable Pain Monitoring

**DOI:** 10.1155/prm/8799914

**Published:** 2026-09-23

**Authors:** Seong Jin Cho, Kwang-Ho Choi, Min Ji Kim, O Sang Kwon, Suk Yun Kang, Hee Young Kim, Yeonhee Ryu

**Affiliations:** ^1^ Korea Institute of Oriental Medicine, Daejeon, Republic of Korea, kiom.re.kr; ^2^ Department of Meridian System and Acupuncture Point, Wonkwang University College of Korean Medicine, Iksan, Republic of Korea; ^3^ Department of Physiology, Yonsei University College of Medicine, Seoul, Republic of Korea, yonsei.ac.kr

**Keywords:** electroencephalography (EEG), functional connectivity, global field synchronization (GFS), pain monitoring, phase synchronization

## Abstract

**Background:**

Integrative pain care emphasizes longitudinal monitoring to personalize multimodal treatment and reduce medication‐related risk. Wearable electroencephalography (EEG) could enable scalable, real‐time neurophysiological tracking but requires connectivity features that are computationally simple and mechanistically valid. Global field synchronization (GFS) is widely used and often interpreted as a global phase‐synchrony index, yet its construct validity remains unclear.

**Objective:**

To evaluate whether GFS validly represents EEG phase synchronization and discuss implications for wearable monitoring in integrative pain management.

**Methods:**

We conducted construct validation using (i) synthetic Fourier‐domain multichannel data designed to generate counterexamples and (ii) empiricalresting‐state EEG data from healthy subjects (32 channels, eyes closed). Phase dispersion was quantified by circular standard deviation (CSD). Simulations assessed GFS–CSD correspondence under rotations (0°–180°) and scaling (1–5). Empirical EEG analyses computed GFS and CSD across 1–50 Hz and evaluated their relationships by frequency band.

**Results:**

In simulations, GFS was invariant to rotations that substantially changed CSD but varied strongly with scaling, whereas CSD changed minimally, indicating that GFS and phase dispersion can dissociate. In empirical resting‐state EEG, an expected inverse relationship between GFS and CSD was not consistently observed across delta, theta, alpha, beta, or gamma bands. At a single frequency, examples showed that similar GFS values could correspond to markedly different phase patterns depending on the point cloud’s location and distribution.

**Conclusions:**

GFS does not generally serve as a valid proxy for EEG phase synchronization, thereby limiting its mechanistic interpretation as a wearable connectivity biomarker. For integrative pain monitoring and decision support, rigorous construct validation should precede clinical translation, and alternative phase‐based indices with real‐world robustness are needed.

## 1. Introduction

Pain is a personal experience influenced by biological, psychological, and social factors, and it cannot be inferred solely from nociceptive activity [1]. In clinical practice, effective pain care therefore depends not only on treatment selection but also on strategic pain monitoring. Consensus recommendations for chronic pain trials emphasize that pain intensity should be assessed alongside domains such as physical and emotional functioning, global improvement/satisfaction, and adverse events—highlighting the need for multidimensional monitoring to guide individualized care [2]. Despite the routine use of numeric rating scales (NRS), pain monitoring remains challenging because pain fluctuates and self‐report is episodic, context‐dependent, and sometimes infeasible. Furthermore, reliance on single‐item scores has been criticized as insufficient to improve the quality of pain management, thereby motivating interest in complementary objective and longitudinal indicators [3]. In parallel, contemporary pain guidelines increasingly encourage patient‐centered reassessment and optimization of nonpharmacologic and multimodal strategies, which benefit from timely feedback on treatment response [4–6]. Digital health and wearable sensing can extend pain monitoring beyond clinic visits by enabling frequent or continuous capture of physiological correlates. However, no gold standard exists for objectively measuring pain; wearable devices should not be interpreted as measuring pain itself, but rather as providing correlational insights through physiological signals that may support responsive monitoring [7]. Among candidate modalities, wearable EEG is particularly appealing because it provides a direct window into neural dynamics that underlie pain perception and modulation, while recent hardware advances have enabled a growing ecosystem of portable and wireless EEG systems suitable for real‐world use [8].

Mechanistically, coordinated activity among local and distributed brain networks is central to pain neurobiology, and oscillatory rhythms provide a substrate for interregional communication and synchronization. Human and rodent studies converge on pain‐related alterations in oscillatory activity—especially in high‐frequency (gamma) rhythms—while emerging evidence also implicates frequency‐specific synchronization patterns in both acute and chronic pain [9]. At the network level, chronic pain has been associated with reorganization of large‐scale functional systems and alterations in phase relationships, supporting the relevance of connectivity and synchrony as candidate biomarkers [10]. Consistently, EEG studies in centralized pain conditions such as fibromyalgia report alterations in long‐distance phase‐based connectivity, suggesting that phase synchronization metrics can capture clinically relevant network dysfunction [11]. In practice, however, assessing brain connectivity often requires resource‐intensive neuroimaging such as fMRI or computationally demanding EEG/MEG connectivity pipelines. Modern phase‐based connectivity approaches frequently involve all‐to‐all pairwise computations and subsequent graph‐theoretic characterization, which can create substantial dimensionality and implementation burdens—especially for real‐time wearable deployment [12]. Therefore, there is a strong interest in connectivity features that are computationally simple yet mechanistically valid.

Global field synchronization (GFS) is attractive because it can be computed from the Fourier‐transformed EEG using a simple covariance‐eigenvalue formulation, yielding a single synchronization value per frequency [13]. GFS has been widely applied across neurological and psychiatric conditions and has been related to resting‐state network organization in simultaneous EEG‐fMRI studies [14–16]. These properties suggest that if GFS is a valid proxy for phase synchronization, it could be integrated with wireless EEG for real‐time monitoring applications within pain care workflows. Despite this promise, the core assumption that GFS reliably represents phase synchronization has not been rigorously verified across plausible signal configurations. The present study, therefore, (i) provides a mathematical review of the relationship between the definition of GFS and phase synchronization and (ii) empirically tests whether GFS tracks phase dispersion using both synthetic Fourier‐transformed data and empirical resting‐state EEG. Our goal is to clarify whether GFS can be safely interpreted as a phase‐synchrony connectivity feature before deploying it as a wearable EEG biomarker in integrative pain monitoring.

## 2. Materials and Methods

### 2.1. Study Design

This study was designed as a methodological validation to examine whether GFS can be considered a valid index of phase synchronization in multichannel EEG. Because GFS has been proposed as a computationally simple frequency‐domain measure of global EEG synchronization, it may appear attractive for real‐time or wearable EEG applications, including longitudinal monitoring in integrative pain care. However, before clinical translation, we must verify whether the mathematical definition of GFS is consistent with the physiological construct that it is assumed to represent. To address this issue, we used two complementary approaches. First, we generated synthetic Fourier‐transformed data to create controlled geometric conditions in which we could directly examine the relationship between GFS and phase synchronization. This allowed us to test whether GFS changes consistently with phase dispersion under rotation and scaling of the same point cloud. Second, we analyzed empirical resting‐state EEG data to determine whether the relationship observed in synthetic data also appears in real‐world EEG signals. In both analyses, we evaluated phase synchronization using circular standard deviation (CSD), with lower CSD indicating stronger phase concentration and higher CSD indicating weaker phase synchronization.

### 2.2. Mathematical Definition of GFS

GFS was calculated according to the original frequency‐domain formulation introduced by Koenig et al. For each EEG epoch, multichannel EEG signals were transformed into the frequency domain using the fast Fourier transform (FFT). At a given frequency *f*, the Fourier‐transformed value of each EEG channel can be represented as a complex value:

(1) Zif=aif+jbif,

where *i* = 1,   … ,  *N* denotes the EEG channel, *N* is the number of channels, *a*_*i*_(*f*) is the real component, and *b*_*i*_(*f*) is the imaginary component. These values can be plotted as points on a two‐dimensional sine‐cosine plane:

(2) xif=aifbif.

Thus, at each frequency, the multichannel EEG data form a point cloud in two‐dimensional space. GFS summarizes the shape of this point cloud by calculating the covariance matrix of the real and imaginary components across channels. The covariance matrix is defined as

(3) Cf=1N−1∑i=1Nxif−x¯fxif−x¯f⊺,

where $\overset{¯}{x}\left(f\right)$ is the centroid of the point cloud. The two eigenvalues of this covariance matrix are denoted as *λ*_1_(*f*) and *λ*_2_(*f*), where *λ*_1_(*f*) ≥ *λ*_2_(*f*) ≥ 0. These eigenvalues correspond to the variance of the point cloud along its major and minor axes. GFS is then defined as

(4) GFSf=λ1f−λ2fλ1f+λ2f.

By this definition, GFS ranges from 0 to 1. When the point cloud is circular or isotropic, the two eigenvalues are similar, and GFS approaches 0. When the point cloud is highly elongated along one dominant axis, one eigenvalue becomes much larger than the other, and GFS approaches 1. Therefore, GFS primarily reflects the geometric anisotropy of the Fourier‐domain point cloud.

### 2.3. Quantification of Phase Dispersion

To evaluate phase synchronization independently of GFS, the phase angle of each channel at frequency *f* was calculated from the corresponding Fourier‐transformed point using the four‐quadrant arctangent function:

(5) θif=atan2 bif, aif.

Because phase is circular, we quantified phase dispersion across channels using the CSD. For each epoch and frequency, the mean resultant length was calculated as

(6) Rf=1N∑i=1Nejθif.

CSD was then defined as

(7) CSDf=−2lnRf.

A smaller CSD indicates that the phase angles are more tightly clustered, corresponding to stronger phase synchronization. A larger CSD indicates that phase angles are more widely distributed, corresponding to weaker phase synchronization.

### 2.4. Expectation of an Inverse Relationship Between GFS and Phase Dispersion

The original interpretation of GFS is based on the geometry of Fourier‐transformed multichannel EEG data in the sine‐cosine plane. Koenig et al. introduced GFS as a frequency‐specific measure of global EEG phase synchronization, interpreting a highly elongated, linear point cloud as indicating strong phase locking across electrodes, whereas a circular point cloud indicated weak or absent phase locking [13]. In this framework, the two eigenvalues of the covariance matrix describe the point cloud’s principal axes. When most channels share a similar phase at a given frequency, their Fourier coefficients are expected to align in a common direction, yielding a dominant eigenvalue and a high GFS value. Conversely, when phases are widely dispersed across channels, the Fourier coefficients are expected to be more isotropically distributed around the sine‐cosine plane, yielding two similar eigenvalues and a low GFS value. This interpretation has been adopted in subsequent clinical EEG studies. For example, GFS has been used as an index of decreased EEG synchronization in Alzheimer’s disease and mild cognitive impairment [14], as a measure related to cognitive decline severity [17, 18], and as an indicator of functional disconnection in Parkinson’s disease and related dementias [15]. GFS has also been applied to anesthesia research, where changes in global field synchrony were interpreted as reflecting the disruption of widespread brain synchronization during altered consciousness [19]. These studies commonly assume that higher GFS values reflect stronger global synchronization, whereas lower GFS values reflect weaker synchronization.

Based on this conventional interpretation, GFS should show a predictable relationship with an independent measure of phase dispersion. If GFS truly represents phase synchronization, then higher GFS values should correspond to more concentrated phase angles across electrodes. In the present study, we quantified phase concentration using CSD. Because lower CSD indicates tighter phase clustering and therefore stronger phase synchronization, the theoretical expectation is an inverse relationship between GFS and CSD:

(8) GFSf↑ ⟺CSDf↓.

Therefore, a valid phase‐synchrony interpretation of GFS requires that GFS and CSD exhibit a negative association in both synthetic and empirical EEG data. If this inverse relationship is not observed, or if GFS and CSD vary independently, then GFS cannot be considered a general proxy for EEG phase synchronization.

### 2.5. Generation of Synthetic Fourier‐Transformed Data

**FIGURE 1:**
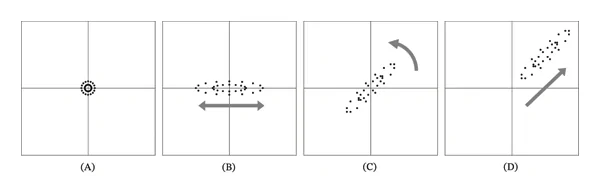
Generation process of synthetic Fourier‐transformed data. (A) Form a circular cloud of points along circles with radii of 1 and 2 centered at the origin. (B) Stretch the cloud along the *x*‐axis. (C) Rotate the cloud around the origin. (D) Translate the cloud into the first quadrant.

Synthetic Fourier‐transformed data were generated to simulate the two‐dimensional point clouds used in the GFS calculation. Figure 1 shows the generation process, which consists of four steps. First, a circular cloud of points was formed along circles with radii of 1 and 2 centered at the origin. To make the synthetic point cloud comparable to the 32‐channel EEG setting, two concentric circular point sets were generated using 16 equally spaced angular samples for each radius:

(9) pr,k=rcosϕksinϕk,r∈12, ,ϕk=2πk16,k=015,…,.

Second, the cloud was stretched along the *x*‐axis to create an elongated distribution. Third, the elongated cloud was rotated around the origin. Fourth, the cloud was translated into the first quadrant to emulate Fourier‐domain point distributions with positive real and imaginary components. These transformations can be expressed as

(10) xr,ks,α,t=RαSspr,k+t,

where

(11) Ss=s001

is the scaling matrix along the *x*‐axis, and

(12) Rα=cosα−sinαsinαcosα.

is the rotation matrix. The translation vector was set as

(13) t=1010.

This synthetic framework allowed us to manipulate the point cloud’s geometry while directly observing how GFS and CSD changed. GFS is expected to be sensitive to cloud elongation; in contrast, CSD is expected to be sensitive to the angular distribution of the points relative to the origin. Therefore, rotation and scaling were used as controlled transformations to determine whether GFS and phase synchronization necessarily covary.

### 2.6. Simulation Analyses

Two simulation analyses were performed to identify counterexamples in which GFS does not properly represent phase synchronization. In the first simulation, the scale factor used to stretch the point cloud was fixed at 5, and the rotation angle was varied from 0^∘^ to 180^∘^. For each rotation angle, we calculated GFS and CSD. This simulation tested whether changes in GFS were due to rotation‐induced changes in the phase distribution. Since rotation preserves the cloud’s shape and therefore the eigenvalue ratio of the covariance matrix, GFS was expected to remain constant. However, because rotation changes the points’ angular positions relative to the *x*‐axis, CSD was expected to vary. The scale factor was chosen to generate a strongly elongated, high‐GFS point cloud. For an initially isotropic point cloud stretched along one axis by a factor *s*, the expected GFS can be approximated as

(14) GFSs=s2−1s2+1.

Thus, when *s* = 5, GFS is 0.923. In the second simulation, the rotation angle was fixed at 45^∘^, and the scale factor was varied from 1 to 5. For each scale factor, we calculated GFS and CSD. This simulation tested whether increasing geometric elongation of the point cloud necessarily corresponds to stronger phase synchronization. Since scaling changes the ratio between the major and minor axes of the point cloud, GFS was expected to increase with the scale factor. However, because the phase angles of the points may change only minimally under this transformation, CSD was not necessarily expected to decrease.

### 2.7. Empirical Resting‐State EEG Data

Empirical resting‐state EEG data were obtained after approval from the Institutional Review Board of the Oriental Hospital of Daejeon University (djomc‐111). Forty‐one healthy adults participated in the study, including 18 males and 23 females, with a mean age of 54 ± 2.9 years. All participants provided written informed consent. EEG was recorded using a 32‐channel EEG system (LXE3232‐RF, LAXTHA, Korea). The ground electrode was attached to the back of the neck, and the reference electrode was attached to the ear. Thirty‐two scalp electrodes were positioned at Fpz, Fp1, Fp2, AF7, AF8, FT7, FT8, T7, T8, TP7, TP8, PO5, PO6, O1, O2, Oz, F3, F4, FC3, FC4, C3, C4, CP3, CP4, P3, P4, AFz, Fz, FCz, CPz, Pz, and POz. We recorded resting‐state EEG for 5 min with eyes closed after a 30‐min rest period. The sampling rate was 512 Hz. For analysis, we randomly extracted thirty 2‐s segments from each participant, yielding a total of 1,230 EEG segments. We applied an FFT to each segment and calculated GFS and CSD at frequencies from 1 to 50 Hz. We then examined GFS–CSD relationships across conventional EEG frequency bands: delta (1–4 Hz), theta (4–8 Hz), alpha (8–13 Hz), beta (13–30 Hz), and gamma (30–50 Hz). This analysis tested whether the theoretical expectation of an inverse relationship between GFS and CSD could be observed in empirical resting‐state EEG data.

### 2.8. Rationale for Not Using Pairwise Phase‐Synchrony Indices as Direct Single‐Epoch Comparators

Established phase‐based connectivity measures such as phase‐locking value (PLV) [20], pairwise phase consistency (PPC) [21], phase lag index (PLI) [22], and weighted phase lag index (wPLI) [23] provide valuable information about phase relationships between signal pairs. However, these measures are fundamentally pairwise metrics that estimate the consistency of phase differences across multiple observations, such as time points, trials, epochs, tapers, or sliding windows. In contrast, GFS is computed as a global index from the Fourier‐domain point cloud of a single epoch at a given frequency. Under this analytical unit, each channel contributes one Fourier coefficient, and each channel pair has only one phase difference. Therefore, pairwise measures cannot be estimated in a directly comparable manner at the same single‐epoch, single‐frequency level. For example, if PLV is calculated from only one phase‐difference observation, it is trivially equal to 1 for every channel pair. PPC is not defined when the number of observations is one. Similarly, wPLI becomes noninformative with a single observation. These measures could be calculated by aggregating multiple epochs or using time‐resolved methods, but doing so would change the analysis from single‐epoch global construct validation to multi‐epoch pairwise or network‐level connectivity analysis. Therefore, the present study focused on CSD, which directly quantifies phase dispersion across the same channel‐wise Fourier points used to compute GFS.

## 3. Results

### 3.1. Rotation Changes Phase Dispersion Without Changing GFS in Synthetic Data

**FIGURE 2:**
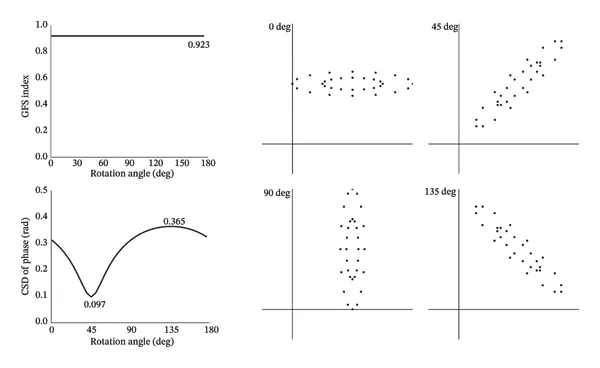
Change in GFS and CSD with respect to the rotation angle. GFS is invariant, whereas CSD changes substantially with rotation.

The first simulation examined whether GFS changes when the same elongated point cloud is rotated around the origin. Figure 2 shows the change in GFS and CSD with respect to the rotation angle. When the scale factor was fixed at 5 and the rotation angle was varied from 0^∘^ to 180^∘^, GFS remained constant at 0.923. This invariance occurred because rotation does not alter the shape of the point cloud or the ratio between the major and minor axes of the covariance ellipse. Since GFS is determined solely by the two eigenvalues of the covariance matrix, the GFS value did not change. In contrast, CSD changed substantially across rotation angles, ranging from 0.097 to 0.365. The lowest CSD was observed when the point cloud was oriented around 45°, where phase angles were relatively concentrated. The highest CSD was observed when the point cloud was oriented around 135°, where phase angles were more widely distributed. Thus, the same point cloud shape yielded different phase dispersion values solely depending on its angular orientation. This result demonstrates that GFS and phase synchronization do not have a one‐to‐one relationship. Although GFS remained fixed because the cloud’s structural anisotropy was unchanged, phase synchronization varied with changes in the angular distribution of points. Therefore, GFS cannot generally be interpreted as a direct measure of phase synchronization.

### 3.2. Scaling Increases GFS Without Increasing Phase Synchronization

**FIGURE 3:**
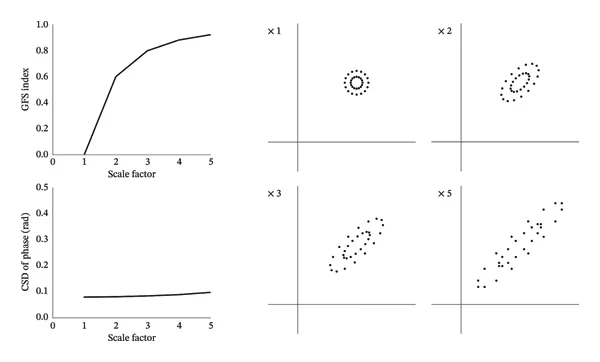
The change in GFS and CSD with respect to the scale factors. GFS changes markedly, whereas the CSD shows only a very small change due to scaling. GFS and phase synchronization do not show a positive correlation.

The second simulation examined whether increasing the point cloud’s elongation necessarily indicates stronger phase synchronization. Figure 3 shows how GFS and CSD change with scale factor. When the rotation angle was fixed at 45^∘^ and the scale factor was increased from 1 to 5, GFS increased markedly from 0 to 0.923. This occurred because scaling along one axis increased the difference between the covariance matrix’s two eigenvalues. As the major‐axis variance increased relative to the minor‐axis variance, the point cloud became more linear, resulting in higher GFS values. However, CSD changed only minimally, increasing slightly from 0.079 to 0.097. This indicates that scaling did not meaningfully concentrate the points’ phase angles. In fact, the slight increase in CSD suggests a small decrease in phase synchronization. Therefore, the large increase in GFS reflected increased geometric elongation of the point cloud rather than increased phase synchrony. This result provides a second counterexample to the common interpretation of GFS. If GFS were a valid phase‐synchrony index, higher GFS values would be expected to correspond to lower CSD values. Instead, GFS increased substantially while CSD remained nearly unchanged, with a slight increase. Therefore, an increase in GFS does not necessarily imply increased phase synchronization.

### 3.3. GFS and CSD Do Not Show a Consistent Inverse Relationship in Empirical Resting‐State EEG

**FIGURE 4:**
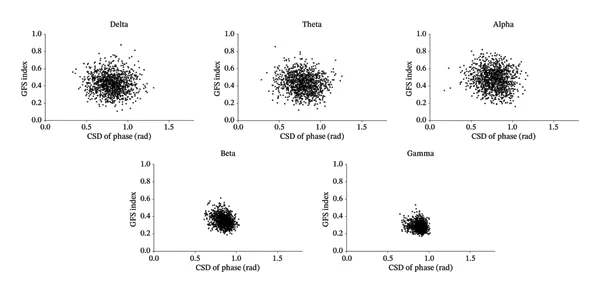
GFS–CSD diagrams in empirical resting‐state EEG data. There is no consistent inverse relationship between GFS and CSD in any band. GFS cannot be assumed to represent phase synchronization in empirical EEG data.

We next examined whether the expected inverse relationship between GFS and CSD could be observed in empirical resting‐state EEG. According to the conventional interpretation of GFS, higher GFS should indicate stronger phase synchronization. Since stronger phase synchronization corresponds to lower CSD, GFS and CSD should be inversely related. However, GFS–CSD diagrams across delta, theta, alpha, beta, and gamma bands did not show a consistent inverse relationship. Figure 4 shows GFS–CSD diagrams in empirical resting‐state EEG data. Across the empirical EEG samples, higher GFS was not reliably associated with lower CSD, and lower GFS was not reliably associated with higher CSD. This indicates that the dissociation observed in the synthetic simulations is not merely a mathematical artifact of artificial data but also appears in real EEG distributions. These findings suggest that empirical EEG point clouds contain diverse geometric configurations in the Fourier domain. In some cases, the cloud shape may align with the phase distribution, making the GFS appear consistent with phase synchronization. In other cases, the point‐cloud location, offset from the origin, or local clustering may produce GFS values that do not match the actual phase dispersion. Therefore, GFS cannot be assumed to provide a stable or generalizable representation of phase synchronization in empirical resting‐state EEG data.

**FIGURE 5:**
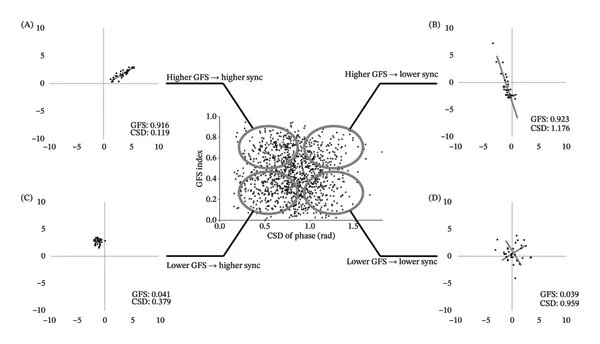
Examples of various relationships between GFS and CSD for 12 Hz. (A) High GFS represents high phase synchronization. (B) High GFS does not represent high phase synchronization. (C) Low GFS does not represent low phase synchronization. (D) Low GFS represents low phase synchronization.

To clarify why GFS sometimes agrees with CSD and sometimes does not, we examined representative examples at 12 Hz. Figure 5 shows examples of four possible relationships between GFS and phase synchronization. First, a high GFS value can correspond to strong phase synchronization. In one example, GFS was 0.916, indicating a highly elongated point cloud. CSD was 0.119, indicating concentrated phase angles. In this case, most points lay near a straight line through the origin, so the cloud’s linear shape also corresponded to a concentrated angular distribution. Under this specific condition, GFS reflected phase synchronization reasonably well. Second, a high GFS value can also correspond to weak phase synchronization. In another example, GFS was similarly high at 0.923 because the point cloud was highly linear. However, CSD was also high at 1.176. This occurred because the linear point cloud was displaced from the origin. Although the covariance structure indicated strong elongation, the actual phase angles were more dispersed. Thus, the high GFS value did not represent strong phase synchronization. Third, a low GFS value can correspond to strong phase synchronization. In one example, GFS was low at 0.041, suggesting a circular point cloud. However, CSD was also relatively low at 0.379, indicating phase concentration. This pattern occurred because the points were densely located within a small region. Although the cloud was not elongated and therefore produced low GFS, the phases were nevertheless concentrated. Fourth, a low GFS value can correspond to weak phase synchronization. In another example, GFS was low at 0.039 and CSD was high at 0.959. In this case, the point cloud was circular, and the points were distributed in multiple directions, producing both low geometric anisotropy and high phase dispersion. Under this condition, GFS and CSD showed the expected inverse relationship. Together, these examples show that GFS is conditionally, but not generally, related to phase synchronization. GFS may reflect phase synchronization when the geometric structure of the point cloud is aligned with the angular distribution of phases, particularly when an elongated cloud passes near the origin. However, when the cloud is displaced, locally clustered, or geometrically circular despite concentrated phases, GFS can produce misleading results. Therefore, GFS should not be interpreted as a general phase‐synchrony connectivity index without additional validation.

## 4. Discussion

This study examined whether GFS can reliably represent phase‐based functional connectivity. Using both synthetic Fourier‐domain point clouds and empirical resting‐state EEG data, we found that GFS and phase dispersion can vary independently, yielding explicit counterexamples and inconsistent relationships across frequency bands.

### 4.1. Why GFS Can Fail as a Phase‐Synchrony Index

GFS is computed from the eigenvalues of the covariance matrix of the point cloud, which primarily encode the cloud’s axis structure, i.e., how “linear” versus “circular” the distribution is. By construction, this representation is insensitive to transformations such as rotation and can be strongly influenced by scaling, whereas phase dispersion depends on the angular distribution of points relative to the reference axis and is sensitive to point location, offset from the origin, and clustering. Consequently, no monotonic mapping between GFS and phase synchronization is guaranteed. The empirical examples further highlight that cloud “shape” alone is insufficient: two clouds with similar elongation can have very different phase concentrations depending on whether the dominant axis passes through the origin or is offset.

### 4.2. Implications for Wearable EEG and Real‐Time Pain Monitoring

Wearable EEG is increasingly feasible in real‐world conditions due to the expanding landscape of wireless devices [8]. In integrative pain care, such wearable neurophysiology could support longitudinal monitoring, remote follow‐up, and treatment personalization—particularly if connectivity can be computed in real time and related to mechanistic targets such as network synchrony. Pain neuroscience emphasizes that coordinated oscillatory activity and interregional synchronization are fundamental to pain processing and modulation [9]. Furthermore, chronic pain conditions show altered network interactions and phase‐related changes at rest, supporting the conceptual relevance of phase‐based connectivity features [10, 11]. A phase synchronization index, as a potential pain‐related monitoring feature, requires that its mathematical definition align with the intended physiological construct. Our results indicate that GFS does not generally quantify phase synchronization. In pain‐monitoring pipelines, this mismatch could lead to incorrect inferences about network‐level coupling, treatment response, or responder phenotypes, especially if GFS values drive adaptive decisions within multimodal care pathways.

The present study should therefore be interpreted as a preventive methodological guide rather than as evidence of widespread misuse of GFS in pain research. The relevance to pain care is prospective and methodological: if EEG‐derived connectivity features are to be used in future wearable or remote monitoring systems, their mathematical definitions must first be aligned with the physiological constructs they are intended to capture. Real‐world wearable EEG conditions may further complicate GFS interpretation. Wearable EEG is more vulnerable to motion artifacts, unstable electrode–skin contact, channel‐specific noise, dry‐electrode impedance changes, environmental noise, and reduced channel density. These factors can alter the Fourier‐domain point cloud by changing its apparent elongation, clustering, offset from the origin, or channel‐wise dispersion. Because GFS is determined by the covariance‐eigenvalue structure of this point cloud, such changes may modify GFS even when they do not reflect meaningful changes in neural phase synchronization. Therefore, before using GFS or any alternative EEG feature for real‐time wearable monitoring, its robustness should be evaluated under realistic wearable acquisition conditions.

### 4.3. Why Standard Connectivity Methods Are Not a Simple Alternative in Wearables

A natural alternative is to use established phase‐based measures such as PLV, PPC, PLI, or wPLI. Nevertheless, whole‐brain implementations often require all‐to‐all pairwise computations that scale rapidly with the number of signals and may be followed by graph‐theoretic summarization, resulting in high‐dimensional and computationally intensive workflows [12, 24]. This complexity is one reason simple global indices such as GFS have been attractive. Our findings, therefore, motivate the development of connectivity summaries that are both valid and computationally tractable, for example, by limiting analysis to a small set of clinically motivated channels, using robust phase‐lag measures, and employing validated global summary statistics.

### 4.4. Recommendations for Future Work in Integrative Pain Care Contexts

Future research should prioritize (1) selecting candidate phase‐based connectivity features with demonstrated construct validity, (2) testing real‐time robustness under wearable artifacts and motion, and (3) evaluating clinical validity in pain cohorts against multidimensional outcomes aligned with pain monitoring standards such as pain intensity, function, emotional distress, sleep, and adverse events [2, 7]. Importantly, the present study focuses on construct validity rather than physiological validation of “connectivity” per se; nonetheless, without construct validity, interpreting any downstream clinical association remains precarious.

### 4.5. Limitations

This study used synthetic data to isolate mathematical properties and empirical resting‐state EEG from healthy participants to test real‐world distributions of Fourier‐domain clouds. We did not analyze pain‐evoked EEG or chronic pain cohorts within this dataset. Therefore, while the motivation is pain monitoring, our conclusions pertain to the general validity of GFS as a phase‐synchrony index. Establishing clinical utility for pain applications will require dedicated datasets and prospective designs. In addition, we obtained the recordings using a laboratory‐grade 32‐channel EEG system rather than a wearable EEG device. Although the study was motivated by future wearable applications, direct validation under wearable conditions remains necessary. The analysis also used whole‐head GFS because GFS is a global field index. This approach does not address regional functional organization, electrode‐pair connectivity, source‐level networks, or volume conduction. Finally, although CSD is appropriate for testing phase dispersion at the same single‐epoch global level as GFS, it does not replace established pairwise connectivity metrics such as PLV, PPC, PLI, or wPLI. Future work should examine how GFS relates to these measures in study designs specifically constructed for pairwise or network‐level analysis.

## 5. Conclusions

GFS is computationally attractive for wearable EEG applications, but it does not generally capture phase synchronization: simulations and empirical resting‐state EEG data demonstrate that GFS and phase dispersion can vary independently and may even exhibit contradictory patterns at the same frequency. These results caution against interpreting GFS as a phase‐synchrony connectivity biomarker in real‐time monitoring pipelines, including those envisioned for integrative pain care. Future work should identify and validate alternative phase‐based connectivity features that are both mechanistically sound and feasible for real‐world wearable deployment.

## Author Contributions

This study was conceived and designed by Seong Jin Cho and Yeonhee Ryu. Simulations were performed by Seong Jin Cho and Suk Yun Kang, and EEG recordings were conducted by Kwang‐Ho Choi, Min Ji Kim, and O Sang Kwon. Data analysis was performed by Seong Jin Cho and Kwang‐Ho Choi. All authors contributed to the interpretation and critical discussion of the results. Seong Jin Cho drafted the manuscript, and Hee Young Kim and Yeonhee Ryu contributed to the manuscript revision.

## Funding

This work was supported by Grant number (No. KSN2512011) from the Korea Institute of Oriental Medicine (KIOM), provided by the National Research Foundation of Korea (NRF) grant funded by the Ministry of Science and ICT (MSIT) (No. RS‐2026‐25478221), conducted at the KIOM (No. NSN2611030).

## Conflicts of Interest

The authors declare no conflicts of interest.

## Data Availability

The data that support the findings of this study are available upon request from the corresponding author. The data are not publicly available due to privacy or ethical restrictions.
